# Long Non-coding RNA DANCR in Cancer: Roles, Mechanisms, and Implications

**DOI:** 10.3389/fcell.2021.753706

**Published:** 2021-10-15

**Authors:** Maoye Wang, Jianmei Gu, Xu Zhang, Jianping Yang, Xiaoxin Zhang, Xinjian Fang

**Affiliations:** ^1^Jiangsu Key Laboratory of Medical Science and Laboratory Medicine, School of Medicine, Jiangsu University, Zhenjiang, China; ^2^Department of Clinical Laboratory Medicine, Nantong Tumor Hospital, Nantong, China; ^3^Department of Orthopedics, Changzhou Traditional Chinese Medicine Hospital, Changzhou, China; ^4^Department of Oncology, Lianyungang Hospital Affiliated to Jiangsu University, Lianyungang, China

**Keywords:** long non-coding RNA, DANCR, cancer, biomarker, diagnosis, therapy, target

## Abstract

Long non-coding RNA (lncRNA) DANCR (also known as ANCR)—differentiation antagonizing non-protein coding RNA, was first reported in 2012 to suppress differentiation of epithelial cells. Emerging evidence demonstrates that DANCR is a cancer-associated lncRNA abnormally expressed in many cancers (e.g., lung cancer, gastric cancer, breast cancer, hepatocellular carcinoma). Increasing studies suggest that the dysregulation of DANCR plays critical roles in cancer cell proliferation, apoptosis, migration, invasion, and chemoresistance *in vitro* and tumor growth and metastasis *in vivo*. Mechanistic analyses show that DANCR can serve as miRNA sponges, stabilize mRNAs, and interact with proteins. Recent research reveals that DANCR can be detected in many body fluids such as serum, plasma, and exosomes, providing a quick and convenient method for cancer monitor. Thus DANCR can be used as a promising diagnostic and prognostic biomarker and therapeutic target for various types of cancer. This review focuses on the role and mechanism of DANCR in cancer progression with an emphasis on the clinical significance of DANCR in human cancers.

## Introduction

LncRNAs are non-coding RNAs (ncRNAs) consisting of longer than 200 nucleotides in length without initiation codon and termination codon. They were initially considered as “junk” transcriptional products without biological functions, thus attracting limited attention among scientists (Anastasiadou et al., 2018). However, in recent decades, large-scale genome-wide sequencing analysis has shown the tissue specificity and essential functions of non-coding RNAs in diverse biological processes (Fico et al., 2019). They can modulate gene expression at epigenetic, transcription, and post-transcription levels (Ma et al., 2019).

LncRNAs also play essential roles in human cancers. The previous studies have indicated that many lncRNAs are dysregulated in cancers (Iyer et al., 2015; Yan et al., 2015). Aberrant expression of lncRNAs is associated with tumor growth (Zhou et al., 2020), metastasis (Liao et al., 2021), angiogenesis (Jin et al., 2020), chemotherapy resistance (Ferretti and León, 2021), and metabolism (Xu et al., 2021). Therefore, lncRNAs are regarded as important regulators of the pathological processes in cancer cells.

**FIGURE 1:**
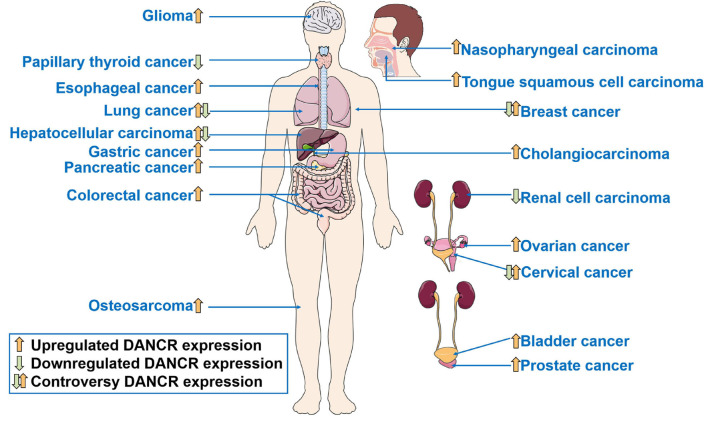
The dysregulation of DANCR expression in human cancers.

**FIGURE 2:**
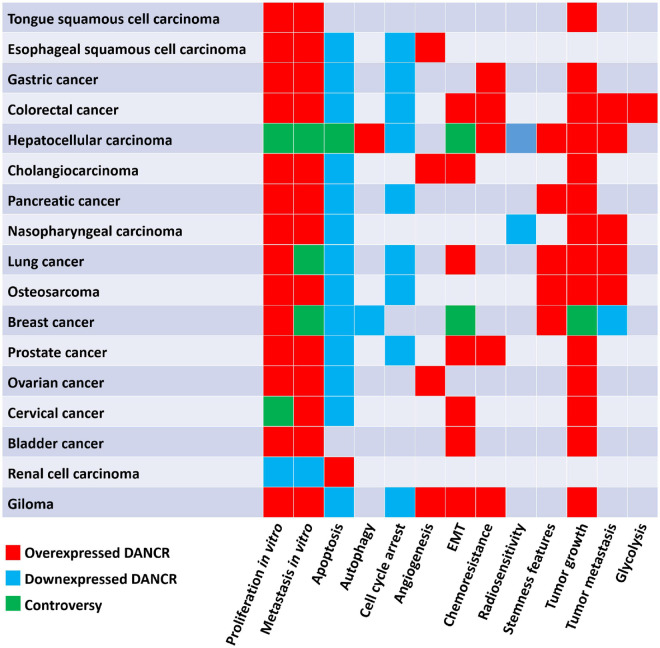
The biological function of DANCR in different cancers.

**FIGURE 3:**
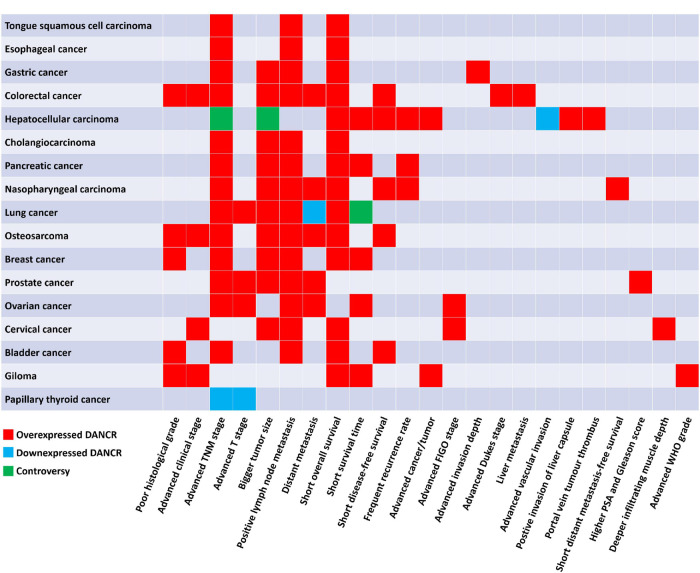
The correlation of DANCR expression level with clinicopathological factors of patients with cancers.

Differentiation antagonizing non-protein coding RNA (DANCR or ANCR), located on human chromosome 4q12, was first reported in 2012 to suppress differentiation of epithelial cells, and then proved to promote the stemness features of hepatocellular carcinoma cells (Kretz et al., 2012; Yuan et al., 2016). Subsequently, in recent decades, many studies have been carried out to understand the function of DANCR in cancer. DANCR is aberrantly expressed in various kinds of cancers (Figure 1). The dysregulation of DANCR expression is closely associated with biological functions (Figure 2) and clinical pathological factors (Figure 3). In this review, we summarized the current evidence regarding the function and underlying mechanism of DANCR in numerous cancers. It is suggested that DANCR serve as a tumor promoter or suppressor, representing a promising cancer biomarker or therapeutic target in various cancers.

## Regulatory Mechanisms for DANCR

**FIGURE 4:**
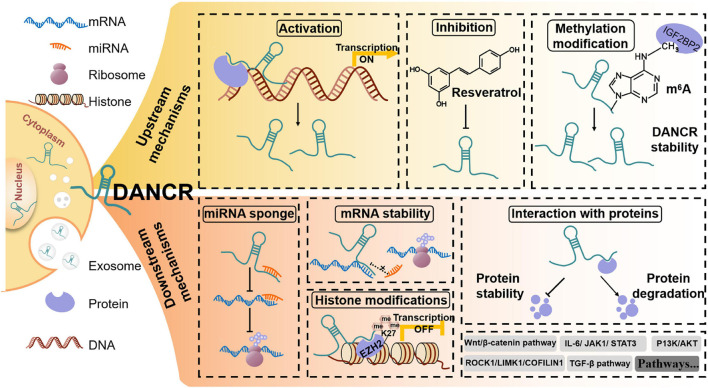
The upstream and downstream regulatory mechanisms for DANCR in cancer. For upstream mechanisms, factors like proteins or drugs can regulate the expression of DANCR. Meanwhile, methylation modification can also regulate DANCR expression. For downstream mechanisms, DANCR can act to sponge miRNAs, stabilize mRNAs and interact with proteins, finally regulating the activation of many signaling pathways.

The downstream regulatory mechanisms for the biological roles of DANCR in cancers are complicated (Figure 4). The competing endogenous RNA (ceRNA) network, initially proposed in 2011, is one popular regulatory mechanism of DANCR in cancers (Table 1; Salmena et al., 2011). As we know, miRNAs could regulate gene expression at the post-transcriptional level by interacting with the 3′UTR region of mRNA via base-pairing (Fabian et al., 2010). Meanwhile, the miRNA recognition elements (MRE) of ceRNA could compete with relevant miRNAs to bind to mRNAs and regulate their expression (Salmena et al., 2011). Up to now, DANCR has been reported to bind to ∼ 50 miRNAs (Table 1).

**TABLE 1:** The molecular mechanisms of DANCR in various cancers.

Cancer	Role	Regulated molecules			Related pathway	References
		miRNA	mRNA	Protein		
Tongue squamous cell carcinoma	Oncogene	miR-135a-5p	KLF8		DANCR/miR-135a-5p/KLF8	Zheng et al., 2019
Esophageal squamous cell carcinoma	Oncogene	miR-33a-5p; miR-4707-3p	FOXC2	ZEB1	DANCR/miR-33a-5p/ZEB1; ZNF750 mutation/DANCR/miR-4707-3p/FOXC2	Zhang C. et al., 2019; Bi et al., 2020
Gastric cancer	Oncogene		MDR1 and MRP1	SALL4; FoxO1	SALL4/DANCR/β-catenin pathway; DANCR/FoxO1; P-gp and MRP1 pathways	Pan et al., 2018; Xie et al., 2020; Xu et al., 2019
Colorectal cancer	Oncogene	miR-577; miR-185-5p; miR-518a-3p; miR-125b-5p; miR-145-5p	HSP27; HMGA2; MDMA; HK2; NRAS; MALAT1	KAT6A; EZH2; QK	DANCR/miR-577/HSP27; DANCR/miR-185-5p/HMGA2; DANCR/miR-518a-3p/MDMA; DANCR/miR-125b-5p/HK2; DANCR/miR-145-5p/NRAS; DANCR/KAT6A acetyltransferase activity; DANCR-EZH2; Doxorubicin/DANCR/QK/MALAT1	Yang et al., 2017; Wang et al., 2018b; Lian et al., 2020; Shi et al., 2020; Sun et al., 2020; Bahreini et al., 2021; Lu et al., 2021; Xiong et al., 2021
Hepatocellular carcinoma	Oncogene	miR-216a-5p; miR-27a-3p; miR-125b-5p; miR-140-3p; miR-222-3p; miR-214, miR-1254, miR-199a, and miR-605; miR−214, and miR−320a	KLF12; LIMK1; HNRNPA1; ATG7; PSMD10; CTNNB1	p-p38/p38, p-ERK1/2/ERK1/2, p-JNK/JNK	DANCR/miR-216a-5p/KLF12; DANCR/miR-27a-3p/LIMK1; ROCK1/LIMK1/COFILIN1; DANCR/miR-125b-5p; MAPK signaling pathway; DANCR/miR-140-3p/HNRNPA1 DANCR/HNRNPA1; DANCR/miR-222-3p/ATG7; DANCR/miR-214, miR-1254, miR-199a or miR-605; DANCR/PSMD10/IL-6/STAT3/DANCR; DANCR/miR-214, miR-320a/CTNNB1	Yuan et al., 2016; Guo D. et al., 2019; Wang J. et al., 2019; Liu et al., 2020a; Wang X. et al., 2020; Wen et al., 2020; Yang et al., 2020
	Tumor suppressor				Wnt/β-catenin signaling pathway	Song et al., 2020
Cholangiocarcinoma	Oncogene	miR-345-5p	TWIST1; FBP1	EZH2	DANCR/miR-345-5p/Twist1; DANCR-EZH2/FBP1	Wang N. et al., 2019; Zhu et al., 2020
Pancreatic cancer	Oncogene	miR-135a; miR-214-5p; miR-33b	NLRP37; E2F2; MMP16	MLL3; IGF2BP2	DANCR/miR-135a/NLRP37; DANCR/miR-214-5p/E2F2; DANCR/miR-33b/MMP16; DANCR/MLL3 (advanced stages); IGF2BP2/DANCR (m6A modified)	Luo et al., 2019; Yao et al., 2019; Hu et al., 2020; Liu et al., 2020b; Tang et al., 2020
Nasopharyngeal carcinoma	Oncogene	miR-4731	HIF1A; PTEN; SOX2; NMT1	NF90/NF45; EZH2; RBM3; STAT3	DANCR- (NF90/NF45)/HIF-1α; DANCR-EZH2/PTEN; Resveratrol/DANCR-EZH2/PTEN; DANCR-RBM3/SOX2; IL-6/JAK1/STAT3; DANCR/miR-4731-5p/NMT1	Ma X. et al., 2018; Wen et al., 2018; Zhang X. et al., 2019; Li et al., 2020; Zhang J. et al., 2020; Ma et al., 2021
Lung cancer	Oncogene	miR-216a; miR-496; miR-1225-3p; miR-758-3p; miR-138; miR-214-5p	MTOR; ERBB2; SOX4; CIZ1; KRAS; HMGA2	EZH2; HMGA2	DANCR/miR-216a; Wnt/β-catenin signaling pathway; DANCR/miR-496/mTOR; DANCR/miR-1225-3p/ErbB2; DANCR/miR-758-3p; DANCR/miR-138/Sox4; DANCR/miR-214-5p/CIZ1; DANCR-EZH2/p21	Lu Q.C. et al., 2018; Wang and Jiang, 2018; Zhen et al., 2018; Bai et al., 2019; Guo L. et al., 2019; Zhang and Jiang, 2019; Chen Y.R. et al., 2020; Yu et al., 2020; Huang et al., 2021
	Tumor suppressor		TGFB1	TGF-β1	TGF-β signaling	Wang et al., 2018a
Osteosarcoma	Oncogene	miR-149; miR-33a-5p; miR-216a-5p; miR-335-5p; miR-1972	MSI2; AXL; SOX5; ROCK1; KRAS and CDKN1B	EZH2; p-p38MAPK	DANCR/miR-149/MSI2; DANCR/miR-33a-5p/AXL; AXL-Akt pathway; DANCR/miR-216a-5p/SOX5; DANCR/miR-335-5p or miR-1972/ROCK1; DANCR-EZH2/p21 and p27; p38MAPK signaling pathway	Jiang et al., 2017; Zhang and Peng, 2017; Wang et al., 2018c; Liu et al., 2019; Pan et al., 2020; Zhang W. et al., 2020
Breast cancer	Oncogene	miR-758-3p; miR-874-3p; miR-4319; miR-216a-5p;	PAX6; SOX2; SOCS3; CD44, ABCG2;	TUFT1; EZH2; RXRA	DANCR/miR-758-3p/PAX6; TUFT1/DANCR/miR-874-3p/SOX2; DANCR/miR-4319/VAPB; DANCR/miR-216a-5p; DANCR-EZH2/SOCS3; DANCR-EZH2/CD44, ABCG2; PI3K/AKT signaling	Sha et al., 2017; Tang et al., 2018; Tao et al., 2019; Jia et al., 2020; Wu et al., 2020; Zhang K.J. et al., 2020; Zhang X.H. et al., 2020
	Tumor suppressor		RUNX2	EZH2	DANCR-EZH2; TGF-β/DANCR/RUNX2	Li et al., 2017b,c
Prostate cancer	Oncogene	miR-135a; miR-34a-5p; miR-214-5p; miR-185-5p	JAG1; LASP1; TIMP2/3	EZH2; MYC, and p21	DANCR/miR-135a; DANCR/miR-34a-5p/JAG1; DANCR/miR-214-5p/TGF-β; DANCR/miR-185-5p/LASP1; AK/PI3K/AKT/GSK3β/snail; DANCR-EZH2/TIMP2/3	Jia et al., 2016; Lu Y. et al., 2018; Ma et al., 2019; Zhao et al., 2019; Deng et al., 2021; Sun et al., 2021
Ovarian cancer	Oncogene	miR-145; miR-214	KLF5	SP1; UPF1	DANCR/miR-145/VEGF; TGF-β/DANCR/miR-214/KLF5; SP1/DANCR; DANCR/UPF1	Lin et al., 2019; Pei et al., 2019; Cui et al., 2020; Huang et al., 2020
Cervical cancer	Oncogene	miR-335-5p; miR-665; miR-145-3p	TGFBR1; FRAT1 and FRAT2	KLF5, ZEB1; FRAT1 and FRAT2	DANCR/miR-335-5p/ROCK1; DANCR/miR-665/TGFBR1; ERK/SMAD pathway; KLF5/DANCR/miR-145-3p/ZEB1; Wnt/β-catenin signaling pathway	Cao et al., 2019; Liang et al., 2019; Tian et al., 2020; Hu et al., 2021
	Tumor suppressor			HIF-1α	DANCR/HIF-1α	Ta et al., 2020
Bladder cancer	Oncogene	miR-149; miR-335	MSI2; VEGF-C	CCND1	DANCR/miR-149/MSI2; IL-11-STAT3 signaling; DANCR/miR-335/VEGF-C	Zhan et al., 2018; Chen et al., 2019; Ping et al., 2021
Glioma	Oncogene	miR-33a-5p; miR-634; miR-216a; miR-33a-5p, miR-33b-5p, miR-1-3p, miR-206, and miR-613	RAB1A; LGR5; AXL; KLF8	EZH2, PTEN	DANCR/miR-33a-5p; DANCR/miR-634/RAB1A; DANCR/miR-216a/LGR5, PI3K/AKT signaling pathway; AXL/PI3K/Akt/NF-κB; Wnt/β-catenin signaling pathway; DANCR-EZH2/PTEN	Li and Zhou, 2018; Ma Y. et al., 2018; Xu et al., 2018; Yang et al., 2018; Wang W. et al., 2019; Cheng et al., 2020

In addition to binding to miRNAs, DANCR could also interact with mRNAs or proteins. For example, DANCR was verified to interact with 3′UTR of CTNNB1 mRNA in hepatocellular carcinoma, thus competitively blocking miRNA site and reversing miRNA-mediated CTNNB1 suppression (Yuan et al., 2016). Similarly, in sorafenib-resistant HCC, DANCR could bind with PSMD10 mRNA to stabilize its expression via blocking the miRNA binding site (Liu et al., 2020a). In the case of binding to proteins, it is interesting that DANCR could regulate both protein stability (Wen et al., 2020) and protein degradation (Xie et al., 2020).

Another function of DANCR is epigenetic regulation. EZH2, a histone methyltransferase of polycomb repressive complex 2 (PRC2), is a common binding protein for DANCR. By interacting with EZH2, DANCR could epigenetically silence target gene transcription. In cholangiocarcinoma, DANCR-EZH2 interaction promoted FBP1 silence by regulating histone methylation of FBP1 promoter and then exacerbating tumorigenesis (Wang N. et al., 2019).

What’s more, DANCR expression is regulated at both epigenetic and transcriptional levels. It was suggested that in gastric cancer cells, DANCR could be activated by SALL4, which could bind to the promoter of DANCR (Pan et al., 2018). In addition, DANCR has been considered as a drug target. In nasopharyngeal carcinoma, resveratrol was verified to suppress cancer progression via DANCR/PTEN pathway (Zhang J. et al., 2020). Moreover, IGF2BP2 protein has been shown to regulate DANCR stability by m^6^A modification (Hu et al., 2020). In conclusion, the upstream and downstream mechanisms of DANCR are multifaceted and play vital roles in human cancers.

## Diagnostic Value of DANCR

It has been verified that DANCR is aberrantly expressed in many cancers. Notably, DANCR is detected in some body fluids, including serum, plasma, and even exosomes (Table 2). This indicates that DANCR may help diagnosis and prognosis in many cancers by non-invasive methods with low cost and real-time monitoring.

**TABLE 2:** The diagnostic and prognostic value of DANCR in cancer.

Cancer	Marker	Sensitivity	Specificity	AUC	Distinction	Source	Detection method	References
Gastric cancer	DANCR	64.6%	67.7%	0.704	GC (*n* = 65)/paired adjacent normal tissues (ANT) (*n* = 65)	Tissues	qRT-PCR	Pan et al., 2018
		72.7%	79.5%	0.816	GC (*n* = 55)/HV (healthy volunteers) (*n* = 39)	Serum		
Colorectal cancer	DANCR	67.5%	82.5%	0.747	CRC (*n* = 40)/HV (*n* = 40)	Serum	qRT-PCR	Shen et al., 2020
	CEA	40.0%	85%	0.623				
	CA199	32.5%	80%	0.573				
	DANCR + CEA + CA199	87.5%	55%	0.812				
	DANCR	67.5%	87.5%	0.745	CRC (*n* = 40)/colorectal polyps (*n* = 10)			
	CEA	–	–	0.555				
	CA199	–	–	0.542				
	DANCR	87.5%	72.5%	0.732	CRC tissues (*n* = 40)/ANT (*n* = 40)	Tissues	qRT-PCR	Bahreini et al., 2021
Hepatocellular carcinoma	DANCR(>−6.2 dCt) DANCR(T/ANT >1.1)	91.2% 88.9%	42.9% 49.1%	0.69 0.74	HCV-HCC recurrence (*n* = 126)/without recurrence (*n* = 57) after curative surgical resection	Tissues	qRT-PCR	Wang S.C. et al., 2020
		83.5%	94.6%	0.88	HCV-HCC recurrence (*n* = 121)/without recurrence (*n* = 56) after curative surgical resection	Exosomes	ddPCR	
		68.3%	85.7%	0.831			qRT-PCR	
	DANCR	83.8%	72.7%	0.868	HCC (*n* = 52)/HV, CHB and cirrhosis (*n* = 94)	Plasma	qRT-PCR	Ma et al., 2016
	AFP	65.4%	77.7%	0.744				
	DANCR	80.8%	84.3%	0.864	HCC (*n* = 52)/CHB and cirrhosis (*n* = 51)			
	AFP	55.8%	76.5%	0.650				
Lung cancer	DANCR	–	–	0.9265	NSCLC (*n* = 72)/HV (*n* = 44)	Tissues	qRT-PCR	Wang et al., 2018a
		–	–	0.8831		Plasma		
Prostate cancer	DANCR	–	–	0.852	PC patients (*n* = 53)/HV (*n* = 47)	Serum	qRT-PCR	Deng et al., 2021)
Ovarian cancer	DANCR	–	–	0.852	OC tissues (*n* = 20)/ANT (*n* = 20)	Tissues	qRT-PCR	Pei et al., 2019
Cervical cancer	DANCR	–	–	0.9073	HPV-negative CSCC (*n* = 38)/HV (*n* = 38)	Tissues	qRT-PCR	Ta et al., 2020
		–	–	0.8740		Serum		
Papillary thyroid cancer	DANCR	85.29%	66.18%	0.8233	PTC tissues (*n* = 76)/ANT (*n* = 76)	Tissues	qRT-PCR	Zhang K. et al., 2019
		81.54%	82.22%	0.8756	PTC (*n* = 49)/HV (*n* = 45)	Tissues (GSE33630)		
		83.33%	91.67%	0.9167	PTC (*n* = 57)/HV (*n* = 9)	Tissues (GSE50901)		
		72.41%	70.83%	0.704	PTC I/II patients (*n* = 57)/III/IV patients (*n* = 19)	Tissues		
Brain tumors	DANCR	91.67%	0.9231%	0.91	Glioma (*n* = 13)/meningioma (*n* = 13)	Tissues	qRT-PCR	Malakootian et al., 2018
		91.67%	0.7778%	0.84	Meningioma (*n* = 13)/pituitary adenoma (*n* = 9)			

Circulating DANCR expression is also positively associated with tissue DANCR level. For example, after HCC surgery treatment, plasma DANCR level decreased dramatically, which indicates that circulating DANCR may drive from tissues (Ma et al., 2016). As expected, the release of RNAs to circulation may reveal disease-associated information of tissue or activation of intercellular signaling pathways (Misawa et al., 2017). Thus, circulating DANCR may have a diagnostic capability. It was reported by Ma et al. (2016) that plasma DANCR had a better diagnostic value than AFP in HCC. As illustrated in Table 2, plasma DANCR (AUC = 0.868) showed a better effect in distinguishing HCC from healthy volunteers (HV), patients with CHB (chronic hepatitis B), and cirrhosis than AFP (AUC = 0.744). Recently, it has been confirmed that DANCR can be transported through exosomes, but the detailed mechanism is unclear. In addition, droplet digital PCR (ddPCR) has been used to detect exosomal DANCR to monitor HCV-HCC recurrence after curative surgical resection, showing a higher AUC (0.88) than qRT-PCR detection (0.831) (Wang S.C. et al., 2020).

These encouraging researches indicate that circulating DANCR may be a novel non−invasive biomarker for cancer diagnosis. However, the lncRNA-based liquid biopsy is still at its early stage. For further application, there is still a long way to go. For instance, standardized sample preparation and reliable endogenous controls need to be considered.

## Function and Mechanism of DANCR in Human Cancers

### Digestive System

#### Tongue Squamous Cell Carcinoma

Tongue squamous cell carcinoma (TSCC) is one of the most common oral cavity malignancies, known for its aggressive biological behavior (Boldrup et al., 2017). Although significant progress has been made in treating TSCC, long-term survival does not improve significantly (Hu et al., 2017). Therefore, it would be beneficial to find promising biomarkers for TSCC detection and treatment.

DANCR expression was upregulated in TSCC cells. *In vitro* studies showed that DANCR could enhance TSCC cell proliferation, migration, and invasion. The molecular mechanism study suggested that DANCR act as a ceRNA to sponge miR-135a-5p and upregulate Kruppel-like Factor 8 (KLF8) expression. Similarly, knockdown of DANCR inhibited tumor growth and KLF8 expression *in vivo*, suggesting that DANCR may play an essential role via miR-135a-5p/KLF8 axis (Zheng et al., 2019). So DANCR may be a diagnostic biomarker and new therapeutic target in TSCC.

#### Esophageal Cancer

Esophageal cancer (EC) is one of the most frequent cancers in the digestive system and ranks the seventh leading cause of death in cancers (Siegel et al., 2021). Though the management and treatment of EC patients have improved, there is still no effective treatment, and 5-year post-esophagectomy survival rates are still poor (Hou et al., 2017; Huang and Yu, 2018). In addition, due to the deficiency of early clinical symptoms, it tends to be diagnosed in advanced stages (Encinas de la Iglesia et al., 2016).

Esophageal squamous cell carcinoma (ESCC) is one histological subtype of EC (Le Bras et al., 2016). Shi et al. (2018) monitored the expression of DANCR in ESCC, and they found that ESCC cell lines and tissues expressed a higher level of DANCR compared with that in adjacent normal counterparts. Its aberrant expression was implicated in accelerating cell proliferation, migration, invasion, and inhibiting apoptosis. Subsequently, Zhang C. et al. (2019) investigated the role of DANCR in ESCC. They found that DANCR sponged miR-33a-5p and upregulated zinc-finger-enhancer binding protein 1 (ZEB1) expression. Recently, Bi et al. (2020) discovered that DANCR was a direct downstream target of a tumor suppressor gene *ZNF750*. The mutations or deletions of *ZNF750* were significantly associated with upregulated DANCR and poor prognosis, especially tumor metastasis in ESCC patients. Their further investigations verified that DANCR could competitively bind with miR-4707-3p and serve as a ceRNA to regulate FOXC2 expression in ESCC, which provides a novel DANCR/miR-4707-3p/FOXC2 pathway regulated by *ZNF750* in ESCC. In summary, DANCR is associated with ESCC progression, and it may act as a novel prognostic and therapeutic target for ESCC.

#### Gastric Cancer

Gastric cancer (GC) is a complex and heterogeneous disease, regarded as the fourth leading cause of cancer-related death (Serra et al., 2019). Traditional clinical indicators such as CEA, CA199, and CA724 are faced with shortcomings in diagnostic sensitivity and specificity. Therefore, there is an urgent need to find a potential marker for GC with higher diagnostic sensitivity and specificity (Cai et al., 2019).

Recent studies have revealed that DANCR expression was upregulated in GC tissues (Hao et al., 2017) and cell lines (Pan et al., 2018) compared to adjacent normal ones. High DANCR expression promoted cell proliferation, migration, and invasion in GC cells. Its expression was positively associated with tumor size, lymph node metastasis, invasion depth, and TNM stage of GC patients (Pan et al., 2018), suggesting that DANCR may correlate to the malignant progression of GC. For further function and mechanism analysis of DANCR in GC, Pan et al. (2018) proved that DANCR was regulated by SALL4 (sal-like protein 4), previously shown as a critical transcription factor to regulate the stemness of GC cells. Then DANCR accelerated GC progression via Wnt/β-catenin pathway (Zhang et al., 2014; Pan et al., 2018). A study by Xie et al. (2020) indicated another mechanism showing that DANCR inhibited FOXO1 expression by promoting its ubiquitination. As a result, M1 macrophage polarization was inhibited, which promoted GC cell invasion and metastasis.

Chemotherapy is the primary therapy for GC patients with unresectable tumors, and cisplatin (DDP) is used as the first-line drug (Zhu et al., 2019). However, DDP resistance is a major obstacle for DDP application. Thus, exploring underlying mechanisms of DDP-resistance development in GC remains an urgent issue (Guo Q. et al., 2019). Xu et al. (2019) found that DANCR was upregulated in DDP-resistant GC cells. Further study showed that the expression of multidrug resistance (MDR) related genes, MDR1 and MRP1, were induced by DANCR in GC cells. Their studies suggest that DANCR is associated with MDR development and may be a potential therapeutic target for GC with MDR.

#### Colorectal Cancer

Colorectal cancer (CRC) is one of the most commonly diagnosed malignant neoplasms among men (Siegel et al., 2019, 2021). Thanks to the improvements in screening tests and treatment, CRC incidence and mortality rates have declined for several years in developed countries (Siegel et al., 2017). However, in some low-income and middle-income regions, CRC incidence and mortality rates are still rising rapidly. Consequently, improvement in treatment options and accessibility is still necessary for economically backward areas (Arnold et al., 2017).

Liu et al. (2015) demonstrated that CRC tissues expressed a higher level of DANCR than adjacent normal ones. In addition, high DANCR expression was associated with worse overall survival and disease-free survival, and further study showed that DANCR might be an independent prognostic factor for CRC.

A recent study by Wang et al. (2018b) showed that miR-577 shared the same binding site for HSP27 (heat shock protein 27) with DANCR. They verified DANCR could enhance CRC cell proliferation and metastasis by acting as a miRNA sponge to promote HSP27 expression. Moreover, in *in vivo* study, the elevation of DANCR promoted tumor growth and liver metastasis of CRC. Another mechanism study by Lian et al. (2020) identified that DANCR could bind with lysine acetyltransferase 6A (KAT6A) and then triggered H3K23 acetyltransferase activity to promote CRC development. Intriguingly, DANCR could also promote the expression of an oncogenic lncRNA MALAT1 via enhancing its RNA stability, which suppressed doxorubicin-induced apoptosis in CRC cells (Xiong et al., 2021). In summary, these studies provided a potential therapeutic target for molecular treatment in CRC.

DANCR was also overexpressed in the serum of CRC patients. Serum DANCR level was positively associated with the TNM stage. Surprisingly, serum DANCR showed a better diagnostic ability than CEA and CA199 and a better performance in distinguishing CRC and colorectal polyps. Moreover, the combination of DANCR with CEA and CA199 showed better sensitivity than the single or double combination (Shen et al., 2020; Table 1), suggesting that DANCR has the potential to be a promising biomarker for CRC patients.

#### Hepatocellular Carcinoma

Hepatocellular carcinoma (HCC) is the most frequent primary liver cancer and ranks the sixth most common neoplasm and the third leading cause of cancer-related death (Forner et al., 2018). Sorafenib is an anti-angiogenic multi-kinase inhibitor for advanced hepatocellular carcinoma. However, the outcomes after therapy are not encouraging (Llovet et al., 2008). So, it is urgent to find other available opinions for treatment. As shown in recent studies, non-coding RNAs are considered promising biomarkers for diagnosis and treatment (Duan et al., 2019).

DANCR was upregulated in HCC cell lines, tissues, plasma and exosomes (Ma et al., 2016; Wang S.C. et al., 2020; Wen et al., 2020). It was implicated that DANCR played an essential role in HCC progression. For example, a high level of DANCR was associated with stemness features (Yuan et al., 2016), metastasis (Wen et al., 2020), and chemotherapeutic drug resistance in HCC (Liu et al., 2020a). In addition, the ROC (receiver operating characteristic) analysis showed that plasma DANCR and exosomal DANCR exhibited good discriminatory ability, suggesting it could be a promising diagnostic marker (Ma et al., 2016; Wang S.C. et al., 2020; Table 2).

DANCR could act as sponges for multiple miRNAs including miR-216a-5p (Wang J. et al., 2019), miR-27a-3p (Guo D. et al., 2019), miR-125b-5p (Yang et al., 2020), miR-140-3p (Wen et al., 2020) and miR-222-3p (Wang X. et al., 2020) in HCC. DANCR could also stabilize PSMD10 (Liu et al., 2020a) and CTNNB1 (Yuan et al., 2016) mRNAs by binding to their 3′UTR region to prevent its degradation by miRNAs. Wen et al. (2020) found that DANCR could not only improve HNRNPA1 expression via DANCR/miR-140-3p/HNRNPA1 axis but also bind to HNRNPA1 protein and inhibit its degradation. However, DANCR was also considered as a tumor suppressor due to the suppression of Wnt/β-catenin signaling pathway in HCC (Song et al., 2020).

In conclusion, DANCR is a crucial factor in HCC progression and may serve as a new marker for HCC diagnosis and treatment.

#### Cholangiocarcinoma

Cholangiocarcinoma (CCA) is a rare but aggressive biliary epithelial tumor (Goyal et al., 2021). Surgery is the best therapeutic option; however, only a minority (35%) of CCA patients are diagnosed early, whereas most of them are diagnosed late due to the “silent” clinical character (Rizvi et al., 2018). Therefore, there is an urgent need for early diagnosis and new treatment methods to help improve CCA survival outcomes.

DANCR was overexpressed in CCA tissues compared with adjacent normal tissues. Its expression level was associated with tumor size, TNM stage, and lymph node metastasis. Moreover, CCA patients with higher levels of DANCR had a lower survival rate. Knockdown of DANCR impeded cell proliferation, migration, invasion, EMT process, angiogenesis, and enhanced apoptosis *in vitro*. miR-345-5p was predicted and verified as a target miRNA of DANCR, which regulated the expression of Twist1 (Twist-related protein 1) in CCA cells (Zhu et al., 2020). Wang N. et al. (2019) showed that DANCR inhibited FBP1 expression epigenetically via interacting with EZH2 and subsequently promoted CCA. In conclusion, DANCR may be a potential biomarker and therapeutic target for CCA.

#### Pancreatic Cancer

Pancreatic cancer is the most life-threatening cancer with the lowest 5-year survival rate (10%) (Siegel et al., 2021). What is worse, early detection for pancreatic cancer is lacking, and the available treatment options are limited (Moore and Donahue, 2019).

DANCR showed higher expression in pancreatic cancer cell lines and tissues. Upregulated DANCR expression was associated with poor prognosis and short overall survival time in patients with pancreatic cancer. Knockdown of DANCR suppressed pancreatic cancer cell proliferation, migration, invasion *in vitro*, and tumor growth *in vivo* (Luo et al., 2019; Yao et al., 2019). Mechanistic studies showed that DANCR could be methylated at the N6 position of adenosine and then be stabilized by the m6A reader protein IGF2BP2 (Hu et al., 2020). In addition, DANCR sponged several miRNAs to regulate the expression of target mRNAs in pancreatic cancer cells (Luo et al., 2019; Yao et al., 2019; Tang et al., 2020). Interestingly, Liu et al. (2020b) found that DANCR could downregulate MLL3 expression to influence pancreatic cancer progression only at a late stage rather than an early stage.

Hence, DANCR was associated with pancreatic cancer development and regarded as a promising target for pancreatic cancer prognosis and treatment.

### Respiratory System

#### Nasopharyngeal Carcinoma

Nasopharyngeal carcinoma (NPC) is not a common cancer type but notable for its distinctive geographical distribution pattern. Most cases occur in the east and southeast parts of Asia (Chua et al., 2016). NPC patients at an early stage or with locoregional advanced disease could be well treated benefiting from the radiotherapy. However, distant metastasis is the primary cause of treatment failure (Yin et al., 2017).

DANCR was upregulated in NPC and promoted NPC cell proliferation, migration, invasion, and inhibited apoptosis *in vitro*. In addition, DANCR knockdown inhibited NPC tumor growth *in vivo* (Hao et al., 2019; Wen et al., 2018). Ma X. et al. (2018) found that DANCR could promote NPC proliferation and radiation resistance via DANCR/PTEN pathway. Interestingly, another research by Zhang J. et al. (2020) found that resveratrol could downregulate the expression of DANCR through this pathway. It validated DANCR as a promising target in NPC therapy.

Hypoxia is known as a target for cancer treatment, including NPC, and intratumoral hypoxia could lead to HIF-1α (hypoxia inducible factor-1α) overexpression (Semenza, 2003; Shan et al., 2018). Wen et al. (2018) found that DANCR overexpression was associated with lymph node metastasis and indicated a poor prognosis in NPC. Further study revealed that DANCR could stabilize HIF-1α mRNA through interacting with NF90/NF45 complex. These data suggest that DANCR might be a potential biomarker and therapeutic target of NPC.

#### Lung Cancer

Lung cancer is the leading cause of cancer-related deaths worldwide, both in men and women. It is reported that nearly a quarter of cancer deaths are related to lung cancer (Siegel et al., 2021). Non-small cell lung cancer (NSCLC) accounts for most types of lung cancer, and lung adenocarcinoma is the most common type of NSCLC. Despite improvements in early detection and standard treatment, NSCLC is often diagnosed at an advanced stage with a poor prognosis (Herbst et al., 2008). Understanding the molecular mechanism of NSCLC may bring better treatment for lung cancer.

Upregulated DANCR expression was detected in NSCLC tissue specimens and cell lines compared with normal counterparts. High level of DANCR was associated with larger tumor size, advanced TNM stage, and lymph node metastasis. Bai et al. (2019) found DANCR could activate EMT and act as a ceRNA to competitively bind to miR-138. And then, Sox4, a vital regulator involving tumor growth and metastasis, was regulated. In addition to miR-138, many other miRNAs have been verified as sponge targets for DANCR (Lu Q.C. et al., 2018; Wang and Jiang, 2018; Zhen et al., 2018; Chen Y.R. et al., 2020; Yu et al., 2020; Huang et al., 2021). Another study by Guo L. et al. (2019) showed that DANCR facilitated carcinogenesis by epigenetically silencing p21 expression via binding to EZH2. Thus, DANCR might be a key regulator of lung cancer progression and used as a promising biomarker.

### Genital System

#### Prostate Cancer

Due to PSA testing and advances in early detection and treatment, the death rate for prostate cancer (PCa) dropped by 51% in the past two decades. However, PCa remains the second leading cause of cancer-related deaths in men, and the high rate of overdiagnosis is widely debated. Androgen deprivation therapy (ADT) is the principal treatment for advanced PCa (Magnan et al., 2015; Siegel et al., 2021). However, the majority of PCa will develop into castration-resistant prostate cancer (CRPC) inevitably over time (Nuhn et al., 2019). Compared with ADT, initial treatment with chemotherapy could improve the survival rate (Kahn et al., 2014; Litwin and Tan, 2017). However, the acquisition of chemoresistance inevitably develops, which is a major reason for therapy failure (Domanska et al., 2012).

Strong evidence has been presented about the oncogenic roles of DANCR in PCa. DANCR was upregulated in PCa tissues and cell lines (Jia et al., 2016; Zhao et al., 2019). Lu Y. et al. (2018) found that DANCR expression was induced by MYC, a common oncogene, which resulted in the reduction of p21, a protein required for cell cycle progression. Enzalutamide, a kind of AR (androgen receptor) inhibitor, was used to treat PCa. However, in some cases, it caused side effects such as PCa metastasis (Lin et al., 2013). DANCR knockdown limited the enzalutamide-induced metastasis. Mechanically, DNACR could inhibit TIMP2/3 expression by binding to EZH2 (Jia et al., 2016). Their studies suggested that DANCR might be a potential target for PCa.

Chemotherapy with Taxol (paclitaxel and its semisynthetic analog docetaxel) is commonly used for CRPC. However, there are obstacles to drug resistance (Gan et al., 2009; Jiang and Huang, 2010). Ma et al. (2019) and Zhao et al. (2019) found that DANCR could function as a sponge to miR-135a and miR-34a-5p, and eventually triggered the resistance to paclitaxel and docetaxel, respectively. Meanwhile, DANCR knockdown could promote the sensitivity of PCa cells to these drugs. Thus, DANCR provides a promising target to improve the effectiveness of chemotherapy for PCa.

#### Ovarian Cancer

Ovarian cancer (OC) is typically diagnosed at an advanced stage and has no effective screening strategy (Matulonis et al., 2016). Searching available biomarkers and developing appropriate targeted therapies are in need (Cortez et al., 2018).

DANCR was detected upregulated in ovarian cancer tissues and cell lines (Lin et al., 2019). Vascular endothelial growth factor (VEGF) is the master regulator of vessel formation, resulting in the growth and metastasis of tumors (Chen et al., 2018). Lin et al. (2019) suggested that DANCR can facilitate angiogenesis by regulating the DANCR/miR-145/VEGF axis in a manner of ceRNA. Additionally, DANCR showed a cancer-promoting property by negatively regulating UPF1. Enhanced UPF1 in OC cells was able to partly reverse the promotion of cell proliferation and migration by DANCR (Pei et al., 2019). Thus, DANCR might serve as a potential therapeutic target for ovarian cancer treatment.

#### Cervical Cancer

Infection of specific types of human papillomavirus (HPV) is the primary biological etiology for cervical cancers (CC). Prophylactic vaccination for HPV provides the most effective method of primary prevention against HPV-related diseases (Kessler, 2017). However, the prognosis of advanced patients with cervical cancer is still poor (Siegel et al., 2021).

Liang et al. (2019) found that the expression of DANCR was aberrantly increased in cervical cancer tissues and cell lines and its high expression was associated with bigger tumor size, advanced FIGO stage, and poorer prognosis. Further functional analysis showed that DANCR could regulate ROCK1 expression by competitively binding to miR-335-5p and promote CC progression. Interestingly, another study by Ta et al. (2020) observed the diagnostic value of DANCR in distinguishing different types of CC. Their further investigation indicated that DANCR downregulated HIF-1α expression and inhibited the growth of HPV-negative CC under hypoxic conditions.

#### Breast Cancer

From Cancer statistics 2021, breast cancer (BC) has become the most common type of malignancy among women worldwide, responsible for 15% of deaths in women (Siegel et al., 2021). TNBC (triple-negative breast cancer), with a poor prognosis, is a subtype of breast cancer that does not express ER, PR, and HER2 (Wang X. et al., 2019). Though endocrine and HER2-targeted therapy have made great progress in recent years, targeted therapies for TNBC remain unsatisfactory (Costa et al., 2018). Therefore, it is necessary to identify new molecular targets for breast cancer therapy.

DANCR was significantly upregulated in TNBC tissues and cell lines compared with normal ones (Tang et al., 2018). Sha et al. (2017) found that DANCR expression was increased in TNBC tissues compared with that in adjacent normal tissues. Patients with higher DANCR expression tended to have worse TNM stage and poorer overall survival (OS). Tao et al. (2019) also found that DANCR knockdown significantly suppressed cancer cell proliferation and invasion, while the opposite phenomena were observed when DANCR was overexpressed. Inhibition of DANCR expression also impaired the growth of breast tumors *in vivo*.

The molecular mechanism analysis showed that DANCR played oncogenic roles by targeting miR-216a-5p as a ceRNA in MDA-MB-231 cells (TNBC cell line) (Tao et al., 2019). In addition, DANCR knockdown was associated with reduced expression of CD44, ABCG2, and ALDH1 in TNBC cells (Sha et al., 2017). Similarly, Tang et al. (2018) showed that DANCR could activate PI3K/AKT signaling through the activation of serine phosphorylation of RXRA by binding of GSK3β and RXRA, which promotes breast cancer progression. Zhang K.J. et al. (2020) verified that DANCR regulated EMT and cancer stemness in BC cells by binding to EZH2, which suppressed SOCS3 (suppressor of cytokine signaling 3) expression. Thus, DANCR was validated as an oncogene in breast cancer, and targeting DANCR may have therapeutic value in BC.

**FIGURE 5:**
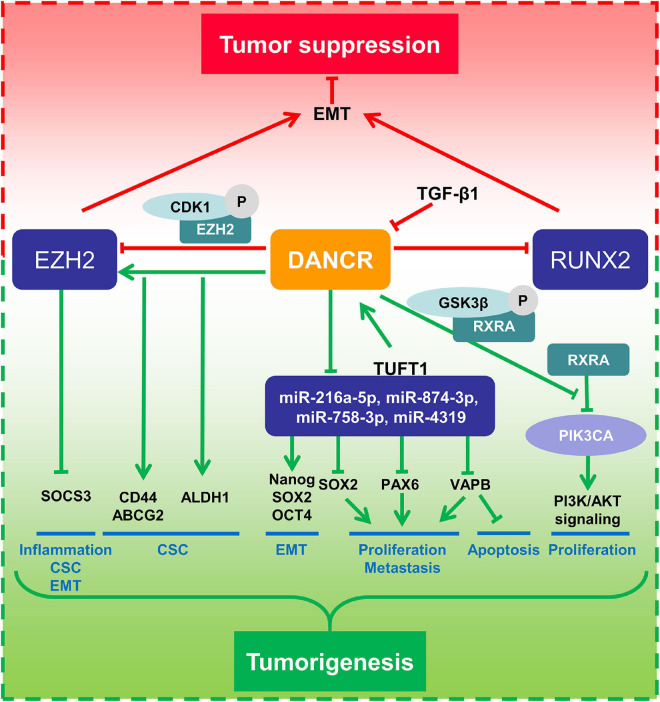
Mechanism of DANCR in breast cancer.

Interestingly, DANCR has also been suggested as a tumor suppressor in BC. Li et al. (2017c) revealed that DANCR expression was downregulated in BC cells as well as tumor tissues. It was verified that DANCR mediated EZH2 degradation and attenuated EMT and metastasis in BC. Another study from the same lab also showed that DANCR could inhibit TGF-β-induced EMT progress and BC metastasis by downregulating RUNX2 expression (Li et al., 2017b; Figure 5).

### Urinary System

#### Bladder Cancer

Bladder cancer (BCa) is among the top ten most common cancer types and the second most common genitourinary malignancy globally, with approximately 550,000 new cases annually (Bhanvadia, 2018; Richters et al., 2019). Diagnosis often occurs too late, particularly in women, due to their misinterpret of hematuria (Grayson, 2017). Thus, it is essential to find potential biomarkers to monitor tumorigenesis, development, and progression in BCa.

Zhan et al. (2018) showed that DANCR was aberrantly upregulated in BCa tissues compared to adjacent normal tissues. Moreover, increased DANCR expression was positively related to higher histological grade and advanced TNM stage. DANCR played as a miRNA sponge to positively regulate the expression of MSI2 (musashi RNA binding protein 2) via sponging miR-149 and subsequently promoted the malignant phenotypes of BCa cells.

Chen et al. (2019) also found that DANCR was significantly upregulated in bladder cancer with lymph node (LN) metastasis. Aberrant expression of DANCR in BCa was associated with LN metastasis and poor prognosis. DANCR guided leucine-rich pentatricopeptide repeat containing (LRPPRC) to stabilize target mRNAs, including IL-11, PLAU, and CCND1. Further investigations indicated that DANCR/LRPPRC/IL-11/STAT3 signaling pathway played an important role in BCa metastasis. These studies suggested that DANCR might be a valuable target for clinical intervention in LN-metastatic BCa.

#### Renal Cell Carcinoma

Renal cell carcinoma (RCC) is one of the most common cancers in the urological system, originating from the renal tubular epithelial system. Surgical resection is an available choice for patients with localized RCC. However, due to the lack of sensitivity to radiotherapy and chemotherapy in RCC, targeted therapy is necessary for individuals without conditions for surgery (Lu et al., 2020).

Jin et al. (2017) found that DANCR was down-regulated in RCC tissues compared to adjacent normal tissues. Overexpression of DANCR caused the suppression of RCC cell proliferation, migration and invasion, and the induction of cell apoptosis. miR-3646 and miR-634 were predicted as the downstream targets of DANCR. However, biological validation studies are needed to confirm.

### Endocrine System

#### Papillary Thyroid Cancer

Papillary thyroid cancer (PTC) is the most prevalent form of thyroid cancer with a rapidly increasing incidence without a concomitant rise in mortality (Jegerlehner et al., 2017; Abdullah et al., 2019). Though the mean survival rate after 10 years is higher than 90%, disease recurrence cannot be ignored (Li et al., 2017a). An efficient and accurate diagnosis of PTC is still needed (Zhang H. et al., 2018).

Zhang K. et al. (2019) found that DANCR expression was lower in PTC tissues compared to that in normal thyroid tissues. Its expression was negatively associated with the clinical stage. ROC curve and AUC suggested the clinical diagnosis value of DANCR in PTC, which indicated DANCR might be a potential marker for PTC diagnosis. However, few mechanism studies about DANCR in PTC have been conducted.

### Nervous System

#### Glioma

Glioma is the most frequent and lethal central nervous system (CNS) tumor occurring both in children and adolescents (Sturm et al., 2017). DANCR expression was significantly higher in glioma cells than in normal human astrocytes (Wang W. et al., 2019). DANCR served as a ceRNA to modulate tumorigenesis, growth and metastasis by sponging miR-33a-5p (Yang et al., 2018), miR-634 (Xu et al., 2018), miR-216a (Wang W. et al., 2019) and miR-135a-5p (Feng et al., 2020) via regulating miR-33a-5p axis, miR-634/RAB1A axis, miR-216a/LGR5 axis, and miR-135a-5p/BMI1 axis, respectively. Li and Zhou (2018) also verified that high expression of DANCR might be a poor prognostic factor in glioma patients. Moreover, Ma Y. et al. (2018) found that DANCR promoted cisplatin resistance via activating AXL/PI3K/Akt/NF-κB signaling pathway through competitively binding with miRNAs, including miR-33a-5p, miR-33b-5p, miR-1-3p, miR-206, and miR-613 in glioma. These studies suggested that DANCR would be a potential biomarker for predicting cisplatin sensitivity and a therapeutic target for enhancing cisplatin efficacy in glioma.

### Motor System

#### Osteosarcoma

Osteosarcoma is one of the most common primary solid malignancies of bone and mainly occurs in adolescence. Though the cure rate for conventional treatment of osteosarcoma is close to 70%, once osteosarcoma has spread to distant organs such as lungs, the survival rates are disappointing (Ritter and Bielack, 2010; Fan et al., 2020). Understanding the molecular mechanisms of osteosarcoma might provide new chances for early diagnosis and targets for therapy.

Increased expression of DANCR could be detected in osteosarcoma tissues and cell lines. Enhanced DANCR was found to have a positive correlation with poor prognostic outcomes (Jiang et al., 2017). DANCR suppression could restrain osteosarcoma progression by inhibiting autophagy (Pan et al., 2020). In recent studies, DANCR was found to function as a ceRNA to promote osteosarcoma progression by sponging miR-33a-5p (Jiang et al., 2017), miR-216a-5p (Pan et al., 2020), miR-149 (Zhang W. et al., 2020), miR-335-5p, and miR-1972 (Wang et al., 2018c). The interaction between DANCR and EZH2 was also found in osteosarcoma, which led to the inhibition of p21 and p27 expression. In conclusion, these studies indicated that DANCR could be utilized as a potential therapeutic target for the treatment of osteosarcoma.

### Other Diseases

Not only in cancer, DANCR could also participate in various biological processes and other diseases. Many research indicated that DANCR might play important role in the differentiation of mesenchymal stem cells (MSCs). Zhang J. et al. (2018) found DANCR was downregulated in human bone marrow-derived MSCs (BD-MSCs) during osteogenic differentiation. Their further investigation revealed DANCR could inhibit proliferation and osteogenic differentiation through p38 MAPK pathway. Similarly, Weng et al. (2021) observed the same function of DANCR in osteogenic differentiation and proposed another mechanism hypothesis. They considered that DANCR might suppress osteogenic differentiation via miR-1301-3p/PROX1 axis (Weng et al., 2021). With a similar situation to BD-MSCs, osteogenic differentiation capacity in periodontal ligament stem cells could also be inhibited by DANCR (Wang Z. et al., 2020). Apart from that, DANCR suppressed vascular smooth muscle cells transforming into osteoblast-like cells, thus attenuating arterial calcification (Zhang X. et al., 2020). Moreover, odontoblast differentiation in human dental pulp cells (Chen et al., 2016; Chen L. et al., 2020) and chondrogenic differentiation in human synovium-derived stem cells (Zhang et al., 2017) was inhibited and promoted by DANCR, respectively. To sum up, our increasing knowledge of DANCR indicated that targeting DANCR may be a novel therapeutic method in many diseases.

## Discussion and Conclusion

Dysregulation of lncRNAs is involved in regulating diverse malignant behaviors of cancer cells, leading to cancer progression and metastasis. It indicates that developing new diagnostic methods and therapeutic options targeting lncRNAs may be a new answer to the fight against cancers. LncRNA-DANCR, a booming researching topic in recent years, has been demonstrated to regulate many cellular functions such as proliferation, apoptosis, EMT, and CSC in various human cancers. The mechanism by which DANCR promotes tumor development is extremely complicated, including serving as a ceRNA for miRNAs, interacting with mRNAs or proteins, activating signaling pathways, and regulating epigenetic modulations. This review depicts a comprehensive picture of the biological roles of DANCR and its underlying mechanisms in cancer.

In most cancers, DANCR was upregulated and acted as an oncogene. However, a minority of studies reported that DANCR functioned as a potent tumor suppressor. Even in the same cancer type, the role of DANCR contradicted with each other (Li et al., 2017c; Zhang X.H. et al., 2020). This may be due to the heterogeneity in different cell lines, clinical sample selection, and experimental design (Table 3). Moreover, some of the confusing results in these studies may be explained by dynamic cancer progression. As in pancreatic cancer, MLL3 was only downregulated by DANCR at an advanced stage (Liu et al., 2020b). In addition, the study of gene expression always focuses on bulk analysis, only showing the information of the dominant cellular subset (Kanzaki and Pietras, 2020). Fortunately, emerging single-cell sequencing can reveal the uniqueness of individual cell and provide individualized therapy for patients (Ding et al., 2020). As shown in Figure 4, the downstream mechanisms are complicated. So when focusing on different targets of DANCR, we may come to different conclusions. These conjectures indicate that more studies on DANCR are needed to further clarify its role in specific cancer types and under distinct conditions.

**TABLE 3:** Information of DANCR in breast cancer from six research articles.

Role of DANCR	DANCR expression in BC cell lines	Compared normal cell line	*In vitro*/*in vivo* validation	Tissue sample size	DANCR expression in tissues	References
Tumorigenesis	↑: MCF7, T47D, MDA-MB-231, MDA-MB-468	Hs578Bst	Both	63 (TNBC)/63 (ANT)	↑: *P* = 0.009	Sha et al., 2017
Tumorigenesis	↑: MCF7, T47D, BT549, MDA-MB-231, MDA-MB-468, MDA-MB-453	MCF10A	Both	60 (TNBC)/10 (normal breast tissue); 60 (TNBC)/15 (Luminal A); 60 (TNBC)/15 (Luminal B)	↑: *P* < 0.001; ↑: *P* < 0.05; ↑: *P* < 0.01	Tang et al., 2018
Tumorigenesis	↑: MCF7, MDA-MB-231	MCF10A	Both	57 (TNBC)/57 (ANT)	↑: *P* < 0.05	Tao et al., 2019
Tumorigenesis	↑: MCF7, T47D, MDA-MB-231, MDA-MB-468	MCF10A	Both	46 (BC)/46 (ANT)	↑: *P* < 0.01	Zhang K.J. et al., 2020
Tumor suppression	↓: MCF7, T47D, BT474, MDA-MB-436, MDA-MB-231, MDA-MB-231HM	MCF10A	Both	32 (BC)/32 (ANT)	↓: *P* < 0.0001	Li et al., 2017c
Tumor suppression	↓: MCF7, T47D, MDA-MB-231HM ↑: BT549	MCF10A	Both	25 (BC)/25 (ANT)	↓ (*P*-value was not provided)	Li et al., 2017b

DANCR is considered as a powerful biomarker not only in discriminating cancer patients from healthy people or patients with benign diseases but also in helping to predict the prognosis for cancer patients. Moreover, the combination of DANCR and other traditional biomarkers may enhance diagnostic efficiency. For cancer treatment, DANCR may be a promising target due to its essential role in cancers. Though DANCR was a promising biomarker and therapeutic target in cancer, more comprehensive and systematic clinical studies and more extensive sample tests are needed to further explore these complex issues. Further studies in DANCR mechanistic investigations and clinical application are still needed. Only after the mechanisms of DANCR in specific cancers have been elucidated can it likely be used for therapeutic purposes.

## Author Contributions

JY, XiZ, and XF conceived the project and supervised the writing. MW and JG wrote the draft of the review. XuZ and XF provided funds and assisted with preparation of the manuscript. All authors are involved in the revision and approved the final version of the manuscript.

## Conflict of Interest

The authors declare that the research was conducted in the absence of any commercial or financial relationships that could be construed as a potential conflict of interest.

## Publisher’s Note

All claims expressed in this article are solely those of the authors and do not necessarily represent those of their affiliated organizations, or those of the publisher, the editors and the reviewers. Any product that may be evaluated in this article, or claim that may be made by its manufacturer, is not guaranteed or endorsed by the publisher.
